# The relationship between sleep quality and physical activity among patients with heart failure: a cross-sectional study

**DOI:** 10.1186/s13102-022-00415-3

**Published:** 2022-02-07

**Authors:** Fatemeh Esnaasharieh, Mahlagha Dehghan, Parvin Mangolian Shahrbabaki

**Affiliations:** 1 MS of Critical Care Nursing, Jahrom University of Medical Sciences, Jahrom, Iran; 2 Nursing Research Center, Razi Faculty of Nursing and Midwifery, Kerman University of Medical Sciences, Haft-Bagh Highway, Kerman, Iran

**Keywords:** Physical activity, Sleep quality, Heart failure

## Abstract

**Background:**

Sleep disorders are one of the most common and annoying problems among patients with heart failure, which decrease their quality of life. Participation in physical activity is one of the most effective methods to reduce sleep disorders; however, few patients participate. This study was conducted to examine the relationship between physical activities and sleep quality among heart failure patients.

**Methods:**

A convenience sample of 100 patients with heart failure referred to rehabilitation centers in southeastern Iran was used in this descriptive cross-sectional study. The Pittsburgh Sleep Quality Index and the Rapid Assessment of Physical Activity (RAPA) were used to collect data. The Spearman correlation coefficient and regression were used to analyze the data. The significance level was < 0.05.

**Results:**

The results revealed that the mean score of sleep quality was 8.74 ± 2.83, with the majority of them (84.47%) having poor sleep quality. The mean score of physical activity was 2.59 ± 1.33, and the majority of them (95.15%) had sub-optimal physical activity. There was a significant and inverse relationship between the total scores of sleep quality and physical activity, and patients’ sleep quality improved while physical activity increased. Physical activity, sex, history of heart surgery, and the stage of illness were found to account for 31% of the variances in patients’ sleep quality.

**Conclusion:**

The results of this study showed better sleep quality among patients who were more physically active. Given that the majority of patients with heart failure suffer from sleep disorders, patients’ knowledge of physical activity should be increased to improve their quality of sleep and quality of life.

## Introduction

In most countries around the world, heart failure is one of the leading causes of disability [1]. It is estimated that by 2030, the global rate of heart disease mortality will have risen to more than 23.3 million people [2]. Patients with heart failure who have a decreased functional capability, dyspnea, and disability face numerous problems, including sleep disorders [3].

Sleep disorder is one of the most common problems among patients with heart failure, and their sleep quality is considered a basic care requirement [4]. Deprivation of good sleep increases the secretion of catecholamine, blood pressure, the amount of oxygen needed by the heart, and ultimately increases the burden on the heart; therefore, it leads to many physical and mental problems as well as a decrease in the quality of life [5]. It seems that lifestyle can affect the symptoms of heart failure such as sleep disorders [6]. However, limitations in lifestyle, social isolation and mood disorders make these patients unable to enjoy adequate and quality sleep [7].

Today, physical activity is one of the simplest and possibly the most inexpensive strategies for improving the lifestyle of cardiovascular patients [8]. Physical activity is any physical movement that requires energy expenditure and is generated by skeletal muscle [9]. Physical activity can moderate risk factors in heart failure patients [10]. Patients can do more activities without becoming tired or short of breath, and improves their quality of life because physical activity improves muscle tone, strengthens the heart muscle and cardiovascular system, which improves the heart's ability to pump, improves circulation and helps the body use oxygen better and increase energy levels [11]. Regular physical activity can also help sleep better, as it can increase sleep duration, improve sleep quality, and reduce the onset of sleep or the time it takes to fall asleep [12]. However, most heart failure patients do not engage in long-term and regular physical activity [13].

According to reports, heart failure patients’ physical activity is on average 16% lower than that of healthy people [14]. It seems that lack of knowledge about self-care and inadequate care at home makes heart failure patients unaware of the importance of physical activity [15]. Even patients with heart failure believe they must rest and do not engage in physical activity after admission to hospital [16]. Therefore, it is important to study the relationship between physical activities and sleep quality and their effects on the quality of life of heart failure patients.

The prevalence of heart failure in Iran is posing a serious challenge to the Iranian health system [17]. Between 29 and 47% of patients are readmitted within 3–6 months of their initial discharge, while 50% of readmissions are avoidable [18]. On the other hand, insufficient physical activity is a concern in Iranian society, particularly among Iranian adults [19]. Thirty to seventy percent of Iranian patients with cardiac diseases, like the rest of the population, do not engage in enough physical activity [20]. Another issue is that 56% of Iranian patients with heart failure suffer from sleep disorders and have to take sleeping pills [21]. A review of the literature shows that physical activity, regardless of its mode and intensity, has increased sleep efficiency, especially in patients on hemodialysis, with diabetes, sleep apnea, obesity, cancer, and rheumatoid arthritis [22–27].

In view of the above, it seems that physical activity among patients with heart failure has been neglected and the problems of patients with heart failure especially sleep disorder and its related factors should be given more attention. In addition, physical activity and sleep habits can be influenced by the culture and customs of societies, which have not been studied in Iran; therefore, this study was performed to investigate the relationship between sleep quality and physical activity among patients with heart failure.

## Method

### Study design and setting

The current research was a cross-sectional descriptive correlational study on patients with heart failure referred to cardiac rehabilitation centers affiliated to Jahrom University of Medical Sciences in southeast Iran with a code of ethics (IR.KMU.REC.1399.269) from Kerman University of Medical Sciences and guideline for reporting observational studies (STROBE). This study lasted from October to January 2020.

### Sample and sampling

Considering correlation coefficient (− 0.391), 95% confidence and 90% test power, 61 samples were calculated, but with an effect size of 1.5, 110 eligible patients admitted to cardiac rehabilitation centers were recruited by the convenience sampling method to minimize the risk of dropout and 103 patients completed the study. The inclusion criteria included patients with stages 2–3 of heart failure diagnosed by a cardiologist [28], patients over the age of 18 who were physically and mentally capable of answering the questionnaire items, and patients in a stable state of the disease. The patients who did not answer to one-third of the questionnaire items and had instable state of the disease were excluded from the study.

### Data collection tools

Data were collected using a demographic information questionnaire, the Pittsburgh Sleep Quality Index (PSQI), and the Rapid Assessment of Physical Activity (RAPA).

### Demographic information questionnaire

patient’s age, sex, marital status, occupation, monthly income, level of education, stage of disease, period of illness, other diseases, medications, weight, addiction, history of heart surgery, other chronic illness, and the number of admissions in the previous 6 months or year were all included in the questionnaire.

### Pittsburgh sleep quality index (PSQI)

This questionnaire is used to assess the quality of sleep and contains 19 items rated on a 4-point Likert scale from 0 to 3. The questionnaire has seven subscales of subjective sleep quality, sleep latency, sleep duration, habitual sleep efficiency, sleep disturbances, use of sleeping medications and daytime dysfunction. The questionnaire total score ranges from 0 to 21 with a score of more than five indicating poor sleep quality. The participant’s score in each item would range from 0 to three, indicating no problem at all, moderate problems, serious problems and very serious problems, respectively. Using Cronbach's alpha, Buysse et al. (1989), who developed and introduced this questionnaire, obtained an internal consistency of 0.83. According to their findings, this scale has a sensitivity of 89.6% and a specificity of 86.5% in the diagnosis of poor sleep (score > 5). Poor sleep quality was diagnosed with a combination of clinical interviews, structured interviews, and polysomnographic data and compared with the standard score above five [29, 30]. In Iran, the validity of this questionnaire was confirmed by content validity. Split-half reliability was used to measure the consistency of the scores of this questionnaire and Cronbach's alpha coefficient was 0.79 [31].

### Rapid assessment of physical activity (RAPA) questionnaire

RAPA assesses the amount and intensity of physical activity and consists of nine items with the response options of yes or no. The first section of the questionnaire defines light, moderate, and vigorous activities for the participants, and the second section includes items about their activities during the week. The RAPA questionnaire consists of seven items that assess the amount, intensity, and duration of physical activity, as well as two items that assess flexibility, strength and the total score of physical activity. The lowest possible score was zero, and the highest possible score was nine. A score of less than six indicates sub-optimal physical activity, while a score of more than six indicates optimal physical activity. To score, choose the question with the highest score with an affirmative response. Any score less than six is sub-optimal. Topolski et al. (2006) used the Spearman’s rank correlation coefficients to validate the physical activity questionnaire and investigate the relationship between physical activity and energy expenditure. They reported RAPA questionnaire with high sensitivity (81%), specificity (69%), and negative predictive value (77%) [28]. In Iran, this instrument was used by Khajavi and Khanmohamadi (2015). The validity of the tool was confirmed by a confirmatory factor analysis. The reliability of the instrument was obtained with Cronbach’s alpha coefficient (0.87) [32].

### Data analysis

Data were analyzed using SPSS25. The demographic and background characteristics of research units, as well as the mean physical activity and sleep quality were described using descriptive statistics (frequency, percentage, mean and standard deviation). The normality of the data distribution was assessed using the Kolmogorov–Smirnov test. Physical activity score did not follow the normal distribution, but distribution of quality of sleep score was normal; therefore, Spearman correlation coefficient was used to examine the relationship between the overall score of sleep quality and its dimensions and physical activity. Multiple linear regression was used to determine predictors of sleep quality and analyze the relationship between a dependent variable (score of sleep quality) and several independent variables. Physical activity and background variables were entered into regression by backward method and the final model was given in the table. The significance level was considered *P* < 0.05.

## Results

The samples had a mean age of 55.98 ± 11.38 years. The mean disease duration among the samples was 7.23 ± 5.03 years. Table 1 lists the remaining demographic characteristics of the patients.

**Table 1:** Demographic characteristic of the patients (n = 103)

Variable	n	%
Sex		
Female	42	40.78
Male	61	59.22
Marital status		
Married	82	79.61
Single/widow/widower/divorced	21	20.39
Education level		
Middle/high school	52	50.49
Diploma	20	19.42
Academic	31	30.09
Income		
< 10 million Rial	16	15.53
10–20 million Rial	21	20.39
20–30 million Rial	33	32.04
> 30 million Rial	33	32.04
Job		
Unemployed	31	30.10
Employed	72	69.90
Stage of illness		
2	23	23.33
3	22	21.36
4	58	56.31
Substance use		
Yes	14	13.59
No	89	86.41
History of heart surgery		
Yes	21	20.39
No	82	79.61
History of other chronic diseases		
Yes	74	71.84
No	29	28.16
Number of admissions		
None	21	20.4
1	37	35.9
2	39	37.9
3 days and more	6	5.8

Variable	Mean	SD
Age (year)	55.98	11.38
BMI	27.10	4.40
Disease duration (year)	7.23	5.03

According to the results, the mean sleep quality of the samples was 8.74 ± 2.83. The majority of patients (84.47%) had poor sleep quality, and among the dimensions of sleep quality, habitual sleep efficiency was the best and sleep duration and sleep disturbances were the worst. Sleep latency (24.27%) was serious in nearly a quarter of patients, and it was very serious in 23.30% of them. The majority of them (48.54%) had no problem with habitual sleep efficiency. 63.11% of them had serious sleep disorders and 43.69% of them had no daytime dysfunction. Subjective sleep quality was relatively good in 49.5% of the patients. However, 38.8% of them had relatively poor subjective sleep quality. 49.51% of the patients slept for 6–7 h, while 37.86% slept for 5–6 h. 44.66% had used sleeping medications at least once a week (Table 2). The results showed that the mean physical activity in patients was 2.59 ± 1.33 and the majority of patients (95.15%) had sub-optimal physical activity (Table 3).

**Table 2:** Sleep quality and its subscales in patients with heart failure (n = 103)

	n	%
Sleep latency		
No problem	19	18.45
Moderate problem	35	33.98
Serious problem	25	24.27
Very serious problem	24	23.30
Habitual sleep efficiency		
No problem	50	48.54
Moderate problem	34	33.01
Serious problem	10	9.71
Very serious problem	9	8.74
Sleep disturbances		
No problem	0	0.0
Moderate problem	38	36.89
Serious problem	65	63.11
Very serious problem	0	0.0
Daytime dysfunction		
No problem	45	43.69
Moderate problem	35	33.98
Serious problem	17	16.50
Very serious problem	6	5.83
Subjective sleep quality		
Very good	8	7.77
Relatively good	51	49.51
Relatively bad	40	38.83
Very bad	4	3.88
Sleep duration		
> 7 h	0	0.0
6–7 h	51	49.51
5–6 h	39	37.86
< 5 h	13	12.62
Use of sleeping medications		
None	57	55.34
Once a week	17	16.50
Twice a week	29	28.16
Total sleep quality		
Normal	16	15.53
Poor	87	84.47

**Table 3:** Physical activity in patients with heart failure

Variable	n	%
Total physical activity		
Sub-optimal	98	95.15
Optimal	5	4.85

According to the results, the total score of sleep quality had a significant and inverse relationship with physical activity, meaning that increasing physical activity improved the sleep quality of heart failure patients. A higher sleep quality score indicates poor sleep quality, while a higher physical activity score indicates optimum physical activity due to the inverse sleep score (Table 4). Among the subscales of sleep quality, subjective sleep quality, habitual sleep efficiency, sleep disturbances, use of sleeping medications and daytime dysfunction had a significant and inverse relationship with physical activity. These results revealed that as physical activity increased, sleep quality improved among the subscales described (Table 5).

**Table 4:** Descriptive statistics of sleep quality, its subscales and physical activity (n = 103)

Variable	M	SD	Median	IQR (Q3–Q1)	Minimum	Maximum
Subjective sleep quality	1.39	0.69	1	(2–1)	0	3
Sleep latency	1.52	1.05	1	(2–1)	0	3
Sleep duration	1.63	0.70	2	(2–1)	1	3
Habitual sleep efficiency	0.79	0.95	1	(1–0)	0	3
Sleep disturbances	1.63	0.48	2	(2–1)	1	2
Use of sleeping medications	0.93	1.21	0	(2–0)	0	3
Daytime dysfunction	0.84	0.90	1	(1–0)	0	3
Total score of sleep quality	8.74	2.83	9	(11–16)	4	16
Physical activity score	2.59	1.33	2	(2–3)	1	7

**Table 5:** Correlation between sleep quality and physical activity (n = 103)

Variable	Physical activity	
	Spearman correlation coefficient	*P* value
Subjective sleep quality	− 0.41	< 0.001**
Sleep latency	− 0.14	0.16
Sleep duration	− 0.15	0.14
Habitual sleep efficiency	− 0.21	0.03**
Sleep disturbances	− 0.30	0.002**
Use of sleeping medications	− 0.004	0.97
Daytime dysfunction	− 0.31	0.002**
Total score of sleep quality	− 0.27	0.005**

**Significant

Based on demographic and background variables, multiple linear regression with backward method was used to predict patients’ sleep quality. The patients’ sleep quality was considered as a dependent variable. Physical activity, age, BMI, period of illness, sex, marital status, income, substance use, history of heart surgery, stage of illness, and number of admissions were all independent variables. Following the selection of the best model, the results revealed that physical activity, sex, history of heart surgery, and stage of disease were predictors of patients’ sleep quality, accounting for 31% of the variances in sleep quality. According to beta coefficients, physical activity and sex had an indirect effect. Patients’ sleep quality has improved in female sex and as a result of increased physical activity. History of heart surgery and stage of disease had a direct impact. Patients’ sleep quality has deteriorated due to a history of heart surgery and increased stage of illness (Table 6).

**Table 6:** Multiple linear regression model for all variables and overall sleep quality (n = 103)

Independent variable	B	SE	Beta	t	Sig	95% confidence	R^2^
Constant value	2.31	0.35	–	6.53	< 0.001	(1.61, 3.01)	0.310
Physical activity	− 0.05	0.02	− 0.21	− 2.13	0.04	(− 0.10,0)	
BMI	− 0.02	0.01	− 0.18	− 1.90	0.06	(− 0.04,0)	
Sex	− 0.14	0.07	− 0.21	− 2.09	0.04	(− 0.27, − 0.01)	
Marital status	0.14	0.08	0.17	1.83	0.07	(− 0.01, 0.30)	
Substance use	− 0.18	0.10	− 0.19	− 1.85	0.07	(− 0.38, 0.01)	
History of heart surgery	0.18	0.08	0.22	2.24	0.03	(0.02, 0.34)	
Stage of disease	0.13	0.04	0.31	2.83	0.01	(0.04, 0.21)	

History of heart surgery: yes = 1, No = 0 Marital status: single/widow/widower/divorced = 1, married = 2

History of substance use: yes = 1, No = 0 Sex: male = 2, female = 1

## Discussion

This study aimed to examine the relationship between sleep quality and physical activity among heart failure patients referred to rehabilitation centers in southeastern Iran.

The results showed that the majority of patients had poor sleep quality. A significant proportion of patients had serious and very serious problems in the subscales of sleep latency, habitual sleep efficiency, sleep disturbances, and daytime dysfunction. A large number of patients had poor subjective sleep quality and inadequate sleep duration, and about half of the patients took sleeping medications. Consistent with the results of the current study, Hajj et al. (2020) in the United States, Azevedo et al. (2015) in Brazil, Jorge-Samitier et al. (2020) in Spain, Lainsamputty et al. (2018) in Indonesia, and Zeighami et al. (2013) and Momayyezi et al. (2015) in Iran all found that sleep quality was poor among heart failure patients [4, 33–37]. According to LEE et al. (2016), the majority of heart failure patients had sleep problems three or more times per week in the past month, and less than one-third took sleeping medications in the last week [38]. Abedi et al. (2012) discovered that half of the patients with cardiovascular disease had a good sleep and rest pattern, which contradicted the results of this study [39]. Edmealem et al. (2020) revealed that poor sleep quality was not common in Ethiopian patients with heart failure. The majority of patients slept for more than seven hours, had high habitual sleep efficiency, and did not use sleeping medications [40]. These differences can be attributed to differences in the support provided by heart failure associations, the amount of training provided during discharge, home care, and differences in lifestyle, socio-demographic and cultural status [38–40].

According to the results, majority of the participants had sub-optimal physical activity. Most studies indicate that a significant number of heart failure patients do not engage in physical activities [13, 41]. Fakharzadeh et al. (2015) conducted a systematic study in Iran and found that the majority of heart failure patients did not engage in enough physical activity [20].

The current study results revealed a moderate significant relationship between sleep quality and physical activity among patients with heart failure, implying that patients who did more physical activity had better sleep quality. Furthermore, as physical activity increased, sleep quality improved in all subscales of subjective sleep quality, habitual sleep efficiency, sleep disturbances, the use of sleeping medications, and daytime dysfunction. On the other hand, when patients get enough sleep, they do not feel drowsy during the day, so they can be more active and focus on their work more. It is particularly important to patients who do more regular physical activity, because good sleep can involve in the healing and repair of their heart and blood vessels and boosts immune system/function [42]. Studies in this field have also shown that patients who have had more physical activity have had better sleep efficiency and duration, regardless of the type and intensity of activity [43, 44]. In this regard, Ash et al. (2020) found that everyday physical activity was associated with either longer sleep at the same night or more physical activity on the next day [45]. DuBose and Hadi (2016) showed the long-term impacts of disturbed sleep include lower physical functioning and increased delirium [46].

Paparrigopoulos et al. (2010) discovered that heart failure patients who engaged in moderate to vigorous physical activity were less likely to suffer from sleep disorders compared with cardiac patients with light physical activity [2]. This study clarifies the importance of physical activity among patients with heart failure and can help health care providers improve sleep quality and quality of life by training patients about physical activity.

The results of the study showed that the variables of physical activity, sex, history of heart surgery, and the stage of illness predicted sleep quality in patients with heart failure. Beta coefficients demonstrated that patients’ sleep quality improved in women and as a result of an increase in physical activity. Patients’ sleep quality decreased due to a history of heart surgery and a high stage of disease. Redeker et al. (2005) discovered a strong relationship between sleep quality and heart failure among patients with chronic heart failure [47]. Alt et al. (2013) demonstrated that women with chronic rhino sinusitis and those with other diseases had poor sleep quality, which was not strongly associated with the disease severity [48]. They believe that the relationship between disease severity and poor sleep is mutual, and disability predicts worse sleep, which in turn may predict quality of life. It is very important to pay attention to physical activity and quality of sleep in heart failure patients with different demographic characteristics because clinically changes to sleep and physical inactivity in cardiovascular inpatients produce delirium, immunosuppression and depression that may lead to readmission and decrease quality of life [49]. Therefore, it is recommended that health care providers pay attention to this important issue in care planning and lifestyle training.

This study had several limitations. Owing to the fact that temporal and seasonal factors affect the quality of sleep, this cross-sectional study cannot assess the patients’ quality of sleep over time and provide objective information about sleep, and it only reflects patients’ perceptions of sleep and its quality. The impact of different cut-off on physical activity and sleep may have different results, which is why further studies are recommended. On the other hand, due to the low value of the correlation coefficient in the relationship between the study variables, the existence of a confounding variable may have caused a false or fake correlation, and finally, reverse causality bias may exist.

## Conclusion

The results showed that the sleep quality of the majority of patients with heart failure in this study was poor and their physical activity was not at a desired level. In addition, patients who were more physically active also had better sleep quality. Therefore, it is recommended to take the necessary measures to improve sleep quality by controlling or eliminating the factors that cause sleep disorders, and also to identify the factors affecting patients’ compliance with physical activity. It seems that improving the methods of increasing physical activity can be a cost-effective care approach in improving the sleep quality of patients with heart failure.

## Data Availability

The datasets used and/or analyzed during the current study are available from the corresponding author on reasonable request.
